# Genome-wide DNA methylation and gene expression patterns of androgenetic haploid tiger pufferfish (*Takifugu rubripes*) provide insights into haploid syndrome

**DOI:** 10.1038/s41598-022-10291-z

**Published:** 2022-05-18

**Authors:** He Zhou, Qian Wang, Zi-Yu Zhou, Xin Li, Yu-Qing Sun, Gu Shan, Xin-Yi Zheng, Qi Chen, Hai-Jin Liu, Wei Wang, Chang-Wei Shao

**Affiliations:** 1 Key Laboratory of Mariculture, Agriculature Ministry, PRC, Dalian Ocean University, Dalian, 116023 China; 2 Key Lab of Sustainable Development of Marine Fisheries, Ministry of Agriculture and Rural Affairs, Yellow Sea Fisheries Research Institute, Chinese Academy of Fishery Sciences, Qingdao, 266071 China; 3 Laboratory for Marine Fisheries Science and Food Production Processes, Pilot National Laboratory for Marine Science and Technology (Qingdao), Qingdao, 266237 China; 4 Dalian Days Industrial Co., LTD, Dalian, 116011 China

**Keywords:** Developmental biology, Genetics, Molecular biology

## Abstract

Androgenesis is an important chromosome set manipulation technique used in sex control in aquaculture. Haploid embryos exhibit haploid syndrome with body abnormalities and even die during early embryonic development. In this study, we used whole genome bisulfite sequencing (WGBS) to investigate the genome-wide DNA methylation profiles in haploid females (1n-X) and males (1n-Y), and diploid females (2n-XX) and males (2n-XY) of tiger pufferfish (*Takifugu rubripes*), an economically important fish in China. A total of 96.32 Gb clean data was produced. Differentially methylated regions (DMRs) were found between haploids and diploids, which may be related to abnormal development and early embryonic death in haploids. There were 3,641 hyper-methylated differentially methylated genes (DMGs) and 2,179 hypo-methylated DMGs in haploid vs. diploid comparisons in both females and males. These DMGs were mainly related to genomic stability maintenance and cell cycle regulation. *slf1*, *actr8*, *gas2*, and *pbrm1* genes were selected to validate the methylation sequencing. After combining the methylation data with the corresponding transcriptome data, we identified several genes, including *guca2a*, *myoc*, *fezf2*, *rprml*, *telo2*, *s100a1*, and *marveld1,* which exhibited differential expression levels modulated by DNA methylation. In conclusion, our study revealed different methylation and expression profiles between haploid and diploid *T.* rubripes for the first time. Several DMGs were identified between different ploidy levels, which may be related to haploid syndrome formation. The results expand the understanding of the effects of ploidy on the early development of teleosts and provide knowledge about target genes and networks to improve the survival rate of haploids.

## Introduction

Sex control is important because of the different features that discriminate between male and female fish. Different features, such as external body color, shape and size are associated with reproduction. A system that includes chromosome set and genome manipulation has been developed to achieve mono-sex biased populations by altering the number or combination of the chromosome set to maximize productivity. Like most vertebrates, male teleosts undergo complete meiosis and spermatogenesis to produce haploid sperms. However, females ovulate physiologically fertile mature eggs arrested at the metaphase of the second meiotic division (MII arrest). Eggs resume the MII arrest and release the second polar body with a haploid set of chromosomes to complete meiosis while activated by sperms. Subsequently, female and male pronuclei are fused to form zygotic nuclei, and mitosis is initiated to continue cell reproduction and differentiation. These features of teleosts facilitate the chromosome set manipulation to produce artificial polyploid and unisexual progeny.

Androgenesis is defined as a chromosome set manipulation technique leading to a uniparental reproduction without any genetic contribution from the maternally derived nucleus^1^. Spontaneous androgenetic teleosts have never been reported in nature^2^. Androgenesis has significant applications in aquaculture, such as rapid generation of inbred lines, all-paternal inheritance, recovering genotypes from cryopreserved sperm of unique or endangered species and studying the effects of mitochondrial genotype on performance. The preparation for artificial androgenesis has two major steps, such as genetic inactivation of egg nucleus and duplication of sperm-derived paternal chromosomes using temperature or hydrostatic pressure shock at an optimal time for inhibiting the cleavage division. Egg nucleus has been genetically inactivated by irradiation with X-ray, γ-ray or ultraviolet (UV) ray^2–4^. These techniques are simple, but egg nuclei, cytoplasm, and various cell organelles can be damaged to a certain extent, thereby causing abnormal development. Recently, Morishima et al*.*^5^ reported that the cold-shock treatment of fertilized eggs of pond loach (*Misgurnus anguillicaudatus*) could successfully induce haploid androgenesis, and then doubled haploid loach were successfully produced based on this technique^6,7^. The feasibility of cold-shock-induced androgenesis has also been reported in large-scale loach (*Paramisgurnus dabryanus*)^8^, zebrafish (*Danio rerio*)^9^, and olive flounder (*Paraichthys olivaceus*)^10^.

Tiger pufferfish (*Takifugu rubripes*) is an economically important fish in China. The mature male has better consumer appeal and economic benefits due to the meat quality and taste. Therefore, it is necessary to develop a safe and efficient method without any irradiation or hormone for all-male production. In our previous study, we used a cold-shock technique to produce androgenetic haploid tiger pufferfish with a proportion of 86.7%^11^. However, the haploid embryos exhibited haploid syndrome, which displayed abnormalities and even died during early embryonic development. As previously shown in pond loach and zebrafish, the egg nucleus could be released along with the second polar body under cold treatment. The sperm nucleus remained in the egg to initiate androgenetic development^5,9^. Nevertheless, no study reported the mechanism of the haploid syndrome in tiger pufferfish. In the present study, we cold-shocked the fertilized eggs of tiger pufferfish to induce haploid androgenesis, and applied whole genome bisulfite sequencing (WGBS) and RNA-seq on androgenesis-derived haploid and zygote tiger pufferfish to investigate the gene expression and methylation changes.

## Materials and methods

### Ethics statement

All experiments in this study were approved by the Animal Study Ethical Committee of Dalian Ocean University, and the experiments were performed according to the Guide for the Care and Use of Laboratory Animals in Dalian Ocean University, Dalian, China. According to the editorial policy for scientific reports, the study was carried out in compliance with the ARRIVE guidelines.

### Haploid androgenic tiger pufferfish production and sample preparation

Parental tiger pufferfish were injected with human chorionic gonadotropin (HCG) 24 h prior to artificial insemination (females: 2500–3000 IU/kg; males: 1250–1500 IU/kg). For haploid androgenic tiger pufferfish production, 8-min-post-fertilization embryos were cold-treated in 4 °C seawater for 60 min and transferred to 20 °C aerated seawater for hatching^11^. The diploid group maintained in 20 °C aerated seawater was prepared as control group. Embryos were developed until 4 day-post-fertilization (dpf) (the stage around eyespots appeared), and thirty embryos were randomly sampled from the cold-treated and control groups, respectively. Each embryo was treated by cold‐dropping with DAPI staining to confirm the chromosome number and to evaluate the cold treatment effect as previously described^12^. The rest embryos were kept until 6 dpf before hatching, and another thirty embryos were randomly sampled from the cold-treated and control groups, respectively. After removing chorion and yolk, each embryo was dissected into three parts, The tail part was used for DNA relative content analysis. The tail fin part was used for genetic sex identification. The remaining part included all tissues representing the whole fish was grinded for homogenization, and divided into two aliquots used for DNA and RNA extraction.

### DNA relative content determination

One-third of each embryo (the tail part) was used for DNA relative content analysis by using Partec PA Flow Cytometry (Munich, Germany) as previously reported^12^.

### Genetic sex identification

For genetic sex identification, one-third of each 6 dpf embryo (the tail fin part) was dissected for genomic DNA (gDNA) extraction using the traditional phenol extraction method. Single nucleotide polymorphism (SNP) in the sex-determining gene, *anti-Müllerian hormone receptor* *type II* (*amhr2*), was used to identify the genetic sex. The locus on the X chromosome was C, and the locus on the Y chromosome was G^13^. Primers, including amhr2-F and amhr2-R, are listed in Table S1. The PCR conditions were as follows: 95 °C for 3 min, 35 cycles of 94 °C for 30 s, 55 °C for 30 s, 72 °C for 40 s, and 72 °C for 5 min. PCR products were gel extracted and ligated to pMD18-T vector for sequencing.

### WGBS

Each of the four groups, including haploid females (1n-X) and males (1n-Y), and diploid females (2n-XX) and males (2n-XY), contained two biological replicates. Each replicate was pooled by three embryos. Total DNA was extracted using QIAamp Fast DNA Tissue Kit (Qiagen, Dusseldorf, Germany) according to the manufacturer's instructions. The quantity of DNA was measured by reading A260/280 ratios by spectrophotometer Agilent 2100 bioanalyzer (Thermo, SC, USA). The fragmented DNA samples were subjected to bisulfite conversion. The Accel-NGS Methyl-Seq DNA Library Kit (Swift, MI, USA) was used for attaching adapters to single-stranded DNA fragments. Briefly, the Adaptase step is a highly efficient, proprietary reaction that was performed for end-repair, tailing of 3’ ends and ligation of the first truncated adapter complement to 3’ ends. The extension step was used to incorporate truncated adapter 1 by a primer extension reaction. The ligation step was used to add the second truncated adapter to the bottom strand. The indexing PCR step increased yield and incorporated full-length adapters. Bead-based SPRI clean-ups (Beckman, IN, USA) were used to remove both oligonucleotides and small fragments and change enzymatic buffer composition. Finally, pair-end 2 × 150 bp sequencing was performed on an Illumina Hiseq 4000 platform (LC Sciences, Houston, TX, USA).

### RNA-seq

Total RNA was extracted using Trizol reagent (Invitrogen, Carlsbad, CA, USA) according to the manufacturer’s instructions. The total RNA quantity and purity were analyzed using Bioanalyzer 2100 and RNA 6000 Nano LabChip Kit (Agilent, CA, USA) with RNA integrity number (RIN) > 7.0. Ten μg of total RNA representing a specific adipose type was subjected to isolate Poly (A) mRNA with poly-T oligo-attached magnetic beads (Invitrogen, Carlsbad, CA, USA). Following purification, the poly(A)- or poly(A) + RNA fraction was fragmented into small pieces using divalent cations under elevated temperature. The cleaved RNA fragments were reverse-transcribed to create the final cDNA library using mRNA-Seq Sample Preparation Kit (Illumina, San Diego, CA, USA) according to the manufacturer’s instructions, and the average insert size for the paired-end libraries was 300 bp (± 50 bp). The paired-end sequencing was performed on an Illumina Hiseq 4000 (LC-Bio, Hangzhou, China).

### Bioinformatics analysis

Cutadapt^14^ and Perl scripts in house were used to remove the reads that contained adapter contamination and low-quality and undetermined bases. Then, sequence quality was verified using FastQC (http://www.bioinformatics.babraham.ac.uk/projects/fastqc/), including the Q20, Q30 and GC-content of the clean data. All downstream analyses were based on clean data of high quality. For WGBS, the reads were mapped to the reference genome FUGU5 (GeneBank assembly accession: No. GCA_000180615.2, https://www.ncbi.nlm.nih.gov/assembly/GCF_000180615.1/) from the National Center for Biotechnology Information (NCBI) database using WALT^15^. After alignment, the reads were deduplicated using SAMTool^16^. For each cytosine site (or guanine corresponding to cytosine on the opposite strand) in the reference genome sequence, the DNA methylation level was determined by the ratio of the number of reads supporting C (methylated) to that of total reads (methylated and unmethylated) using per script in house and MethPipe^17^. Differentially methylated regions (DMRs) were calculated by R package-MethylKit^18^ with default parameters (1000 bp slide windows, 500 bp overlap, methylation difference > 20%, sliding linear model (SLIM) corrected *p*-value (*q* value) < 0.05). Differentially methylated genes (DMGs) were defined as genes containing DMRs in their putative promoter regions (TSS − 2.2 kb to + 500 bp). GO (Gene Ontology) and KEGG (Kyoto Encyclopedia of Genes and Genomes) enrichment analyses were performed on the differentially expressed unigenes by Perl scripts in house with a threshold of FDR adjusted *p*-value < 0.05.For RNA-seq, de novo assembly of the transcriptome was performed with Trinity 2.4.0^19^. Trinity grouped transcripts into clusters based on shared sequence content. A transcript cluster was referred to gene. The longest transcript in the cluster was chosen as the gene sequence (aka unigene). All assembled unigenes were aligned against the non-redundant (NR) protein database (http://www.ncbi.nlm.nih.gov/), Gene Ontology (GO) (http://www.geneontology.org), SwissProt (http://www.expasy.ch/sprot/), Kyoto Encyclopedia of Genes and Genomes (KEGG) (http://www.genome.jp/kegg/), and EggNOG (http://eggnogdb.embl.de/) databases using DIAMOND^20^ with a threshold of Evalue < 0.00001. Salmon 1.4.0 was used to determine the expression levels for unigenes by calculating transcripts per million mapped reads (TPM)^21,22^. The differentially expressed unigenes were selected with log2 (fold change) > 1 or log2 (fold change) < -1 and statistical significance (*p* value < 0.05) by R package edgeR^23^. Then, GO and KEGG enrichment analyses were performed on the differentially expressed unigenes by Perl scripts in house.

### Bisulfite sequencing-PCR (BS-PCR)

To check the reliability of WGBS, four DMGs were selected to perform BS-PCR verification, including SMC5-SMC6 complex localization factor 1 (*slf1*), actin-related protein 8 (*actr8*), growth arrest-specific protein 2 (*gas2*) and polybromo 1 (*pbrm1*). Pooled DNA samples from the same family were sodium bisulfite-modified following the manufacturer’s instructions of EZ DNA Methylation-Gold Kit (ZYMO, Irvine, CA, USA). The primers for BS-PCR were identified by online MethPrimer design software (http://www.urogene.org/methprimer/) (Table S1). PCR was performed using TaKaRa EpiTaq HS (Takara, Shiga, Japan) in accordance with the manufacturer’s instructions. The amplified products were purified and cloned into pEASY-T1 vector, and at least 20 clones were randomly selected for sequencing.

### Quantitative real time-PCR (qRT-PCR)

To check the reliability of RNA-seq, six DEGs were randomly selected to perform qRT-PCR verification, including transcription factor AP-2 epsilon (*tfap2e*), oligodendrocyte transcription factor 2 (*olig2*), proline-rich transmembrane protein 1 (*prrt1*), cAMP-regulated phosphoprotein 19 (*arpp19*), MAF BZIP transcription factor K (*mafk*), and WD repeat domain 1 (*wdr1*). The primers for qRT-PCR were shown in Table S1. The qRT-PCR was performed using SYBR Premix Ex Taq™ II (Tli RNaseH Plus) (Takara, Shiga, Japan) according to the manufacturer’s instructions. The PCR conditions were as follows: 95 °C for 30 s, followed by 40 cycles of 95 °C for 5 s and 60 °C for 30 s. All samples were run with triplicate. The relative expression levels were calculated according to the 2^−∆∆Ct^ method. Statistical significance was determined by one-way analysis of variance (ANOVA). Significance was set at *p* < 0.05.

## Results

### Determination of diploid and cold-treated haploid tiger pufferfish

**Figure 1:**
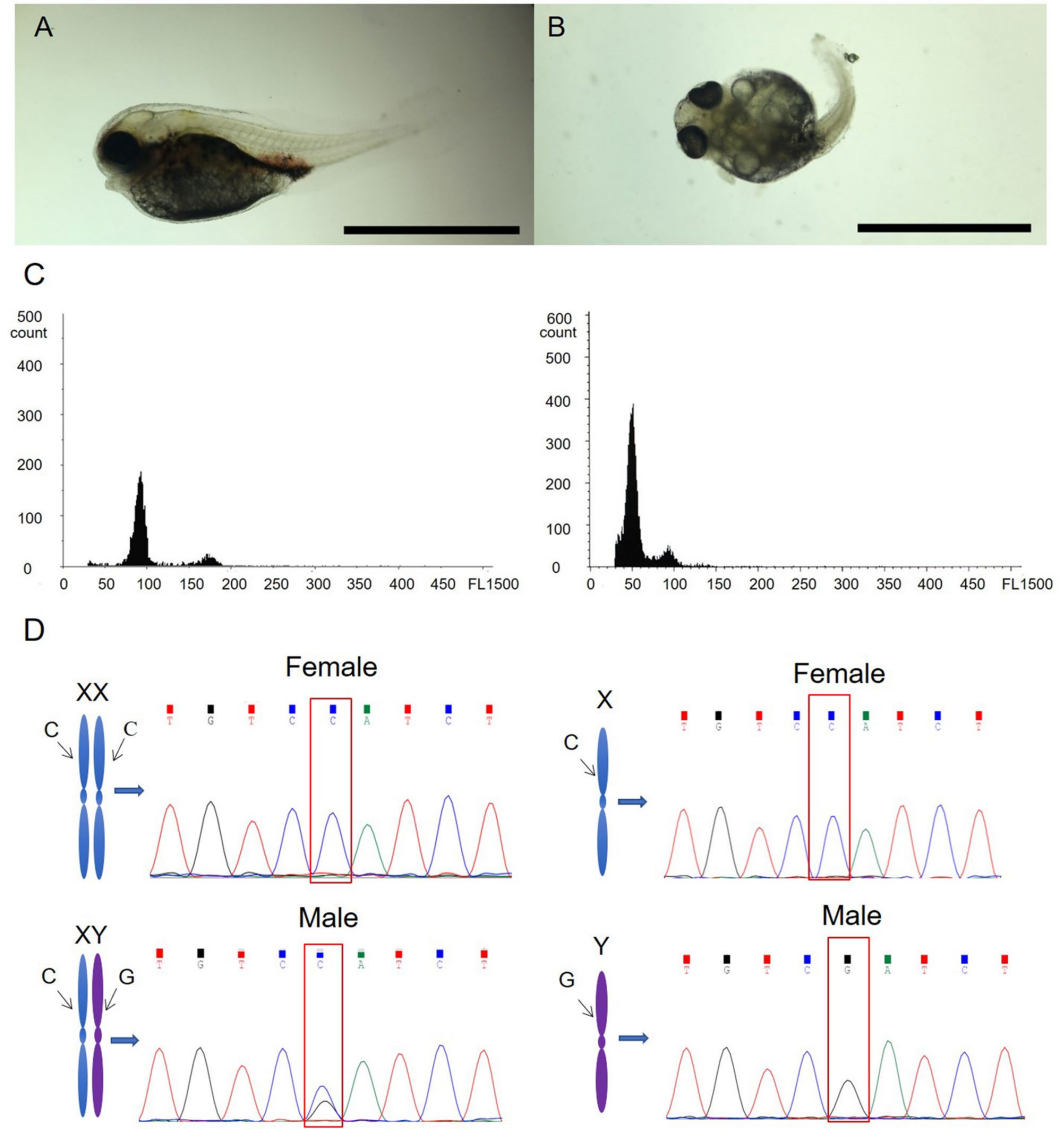
Sex identification of haploid and diploid tiger pufferfish (*T. rubripes*). Morphological observation of a representative fish from diploid group (**A**) and haploid group produced by cold-shock (**B**). Bar = 0.5 mm. (**C**) Flow cytometry results of diploid (left panel) and cold-treated haploid (right panel) tiger pufferfish. (**D**) Genetic sex identification of tiger pufferfish. SNP genotypes of the *amhr2* gene in female and male diploids (left panel) were CC and CG, respectively. SNP genotypes in female and male haploids (right panel) were C and G, respectively.

Embryos from the cold‐shock group showed haploid chromosome number 1n = 22, while embryos showed 2n = 44 in the control group. At 6 dpf, embryos in the control group had normal appearance (Fig. 1A), while most embryos in the cold-shock group showed abnormalities, including spinal curvature, tail malformations, insufficiency of blood circulation, etc. (Fig. 1B). The DNA content of cold-treated haploid tiger pufferfish was half of that of diploid tiger pufferfish (Fig. 1C). The specific SNP locus in the *amhr2* gene was sequenced. The genotypes of female and male diploid tiger pufferfish were CC and CG, respectively. In haploid tiger pufferfish, the genotypes of genetic female and male haploid were C and G, respectively (Fig. 1D).

### WGBS data

To evaluate the DNA methylation patterns across different chromosome ploidy and sex of tiger pufferfish, we performed WGBS on genomic DNA extracted from haploid females (1n-X) and males (1n-Y), and diploid females (2n-XX) and males (2n-XY). A total of 96.32 Gb clean data were produced from the eight libraries, which yielded an average depth of 33 × per strand for each sample (Table S2). The data were uploaded to NCBI Sequence Read Archive (SRA accession: PRJNA535391).

**Figure 2:**
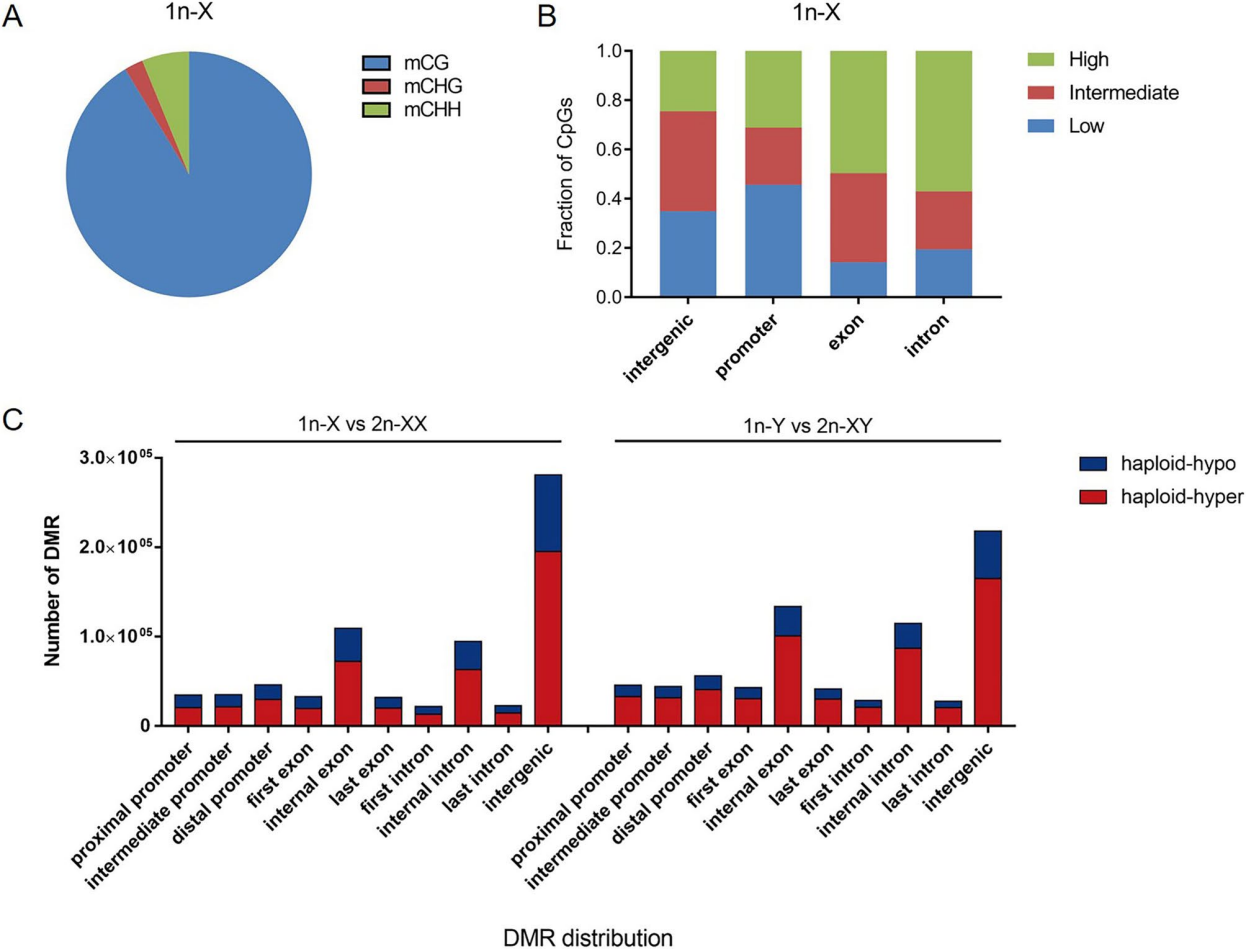
DNA methylation of haploid and diploid tiger pufferfish (*T. rubripes*). (**A**) Percentages of mCs in the mCG, mCHG and mCHH contexts (1n-X was shown). (**B**) Methylation level of genome regions (1n-X was shown). (**C**) Distribution of differentially methylated regions (DMRs) across the genome.

An average of 33.29% of genomic cytosines (Cs) was covered, and the average percent of mCs was 10.80%. Among the mCs, 90.95%, 2.58%, and 6.47% were mCG, mCHG, and mCHH types, respectively (Fig. 2A, Table S3). The mCG sites were focused on the subsequent analyses. Then, the methylation status of CGs was analyzed in various genomic elements. The high methylation level CGs (methylation level > 0.75) were enriched in exons and introns, whereas low methylation level CGs (methylated level < 0.25) were enriched in gene promoters and intergenic regions (Fig. 2B). The chromosome methylation status of mCGs was analyzed. The highest average mCG level was observed on chromosome 1 (74.32%, 72.15%, 72.72%, and 71.41% for 1n-X, 1n-Y, 2n-XX, and 2n-XY, respectively), and the lowest average mCG level was observed on chromosome 18 (28.89%, 28.00%, 28.15%, and 27.55% for 1n-X, 1n-Y, 2n-XX, and 2n-XY, respectively) (Fig. S1).

### Analysis of DMRs

**Figure 3:**
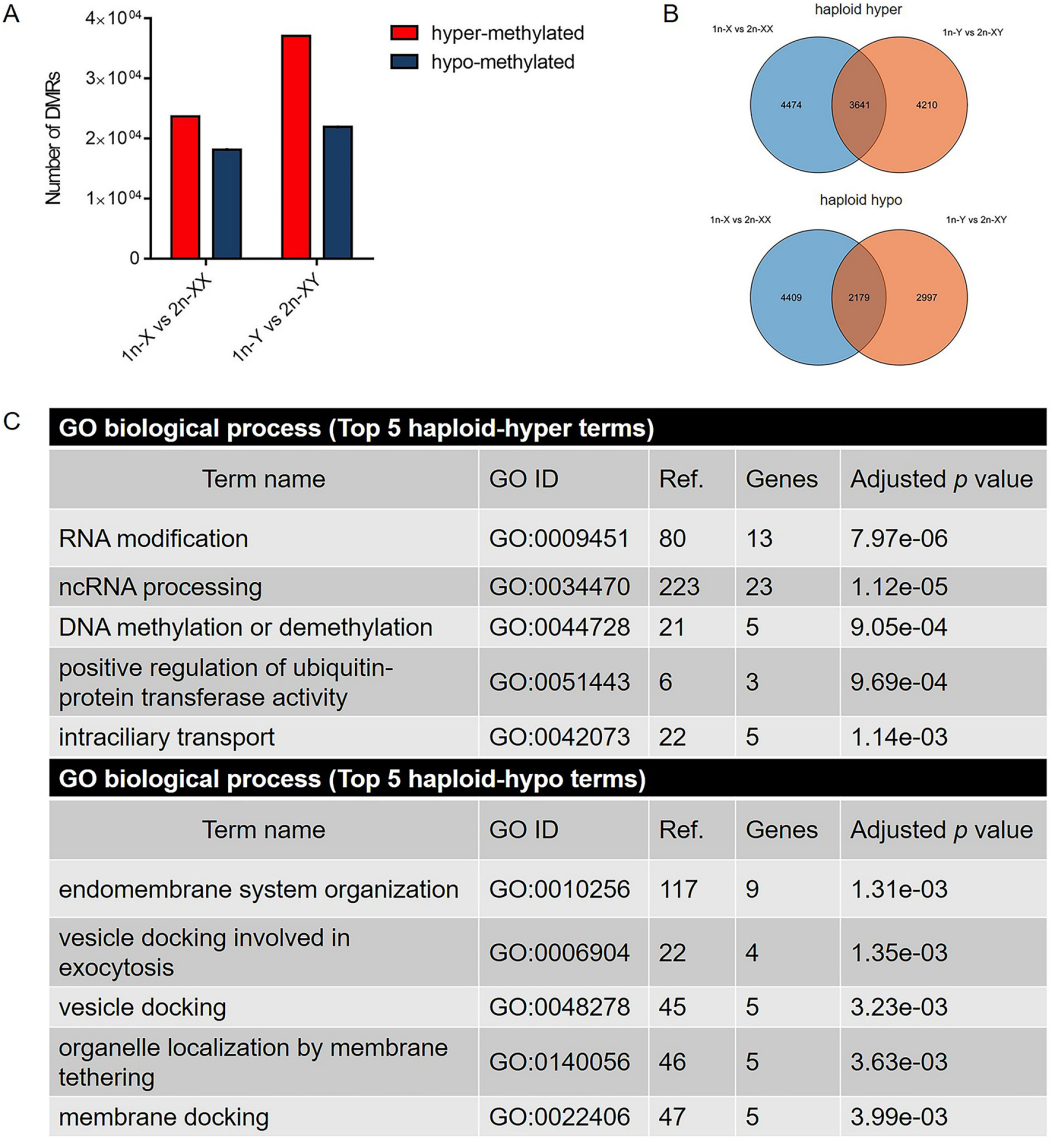
1n-X vs. 2n-XX and 1n-Y vs. 2n-XY comparisons in tiger pufferfish (*T. rubripes*). (**A**) Number of promoter region DMRs between haploid and diploid female and male tiger pufferfish. (**B**) Venn diagram of haploid hyper-methylated/haploid hypo-methylated DMGs between males and females, respectively. (**C**) Top 5 enriched GO biological processes of haploid hyper-methylated/ haploid hypo-methylated DMGs shared in female and male tiger pufferfish.

Pair-wise comparisons were applied to detect DMRs between haploid and diploid tiger pufferfish (1n-X vs. 2n-XX and 1n-Y vs. 2n-XY). In 1n-X vs. 2n-XX comparison, there were 470,641 haploid hyper-methylated and 240,996 haploid hypo-methylated DMRs, respectively. In 1n-Y vs. 2n-XY comparison, there were 560,100 haploid hyper-methylated and 194,484 haploid hypo-methylated DMRs, respectively. The distribution of the DMRs was most enriched in the intergenic region with a range of 27.42–41.50%. The DMRs distribution in promoters, exons, and introns were 15.34–21.35%, 23.87–29.07%, 19.29–22.97%, respectively (Fig. 2C). We then focused on the DMRs located in gene promoter regions (TSS − 2.2 kb to + 500 bp) (methylation difference > 20%, *q* value < 0.05) to identify the corresponding differentially methylated genes (DMGs). In 1n-X vs. 2n-XX comparison, there were 23,725 haploid hyper-methylated and 18,192 haploid hypo-methylated DMRs, corresponding to 8115 and 6588 protein coding DMGs, respectively. In 1n-Y vs. 2n-XY comparison, there were 37,093 haploid hyper-methylated and 21,959 haploid hypo-methylated DMRs corresponding to 7851 and 5176 protein coding DMGs, respectively (Fig. 3A,B).

To evaluate the functions of these DMGs, GO and KEGG pathway enrichment analyses were used. DMGs between haploid and diploid females were significantly enriched in GO terms such as RNA methyltransferase activity, RNA modification, RNA methylation, and ncRNA processing (*p* < 0.05) (Fig. S2A). In males, the significantly enriched GO terms were included DNA binding, sequence-specific DNA binding, MAP kinase activity, RNA methyltransferase activity, and DNA-binding transcription factor activity (*p* < 0.05) (Fig. S2B). Also, DMGs between haploid and diploid were significantly enriched in olfactory transduction, tight junction, lysine degradation, etc. in females, and spliceosome, ubiquitin mediated proteolysis, progesterone-mediated oocyte maturation, etc. in males (*p* < 0.05) (Fig. S2C,D).

When compared DMGs between 1n-X vs. 2n-XX and 1n-Y vs. 2n-XY, 3,641 DMGs were hyper-methylated in haploid vs. diploid comparisons in both females and males, and 2179 DMGs were hypo-methylated in haploid males and females (Fig. 3B). GO enrichment analysis revealed that the shared haploid hyper-methylated DMGs were significantly enriched in GO terms, such as RNA modification, ncRNA processing, DNA methylation or demethylation, positive regulation of ubiquitin-protein transferase activity, and intraciliary transport. Haploid hypo-methylated DMGs were significantly enriched in GO terms such as endomembrane system organization, vesicle docking involved in exocytosis, vesicle docking, organelle localization by membrane tethering, and membrane docking (Fig. 3C). The GO terms of mitotic sister chromatid segregation and positive regulation of mitotic cell cycle phase transition were also significantly enriched in haploid hyper-methylated DMGs (*p* < 0.05) (Table S4). Several DMGs related to genomic stability maintenance and cell cycle changed the methylation levels between haploids and diploids. For example, *slf1* and *actr8* were hyper-methylated in haploid females and males. *gas2* and *pbrm1* were hypo-methylated in haploid females and males. The validation of the DMG data was conducted on the four genes, and the results were in agreement with the WGBS data (*R*^*2*^ = 0.974. *p* < 0.001) (Fig. S3).

### Transcriptome sequencing and assembly

**Figure 4:**
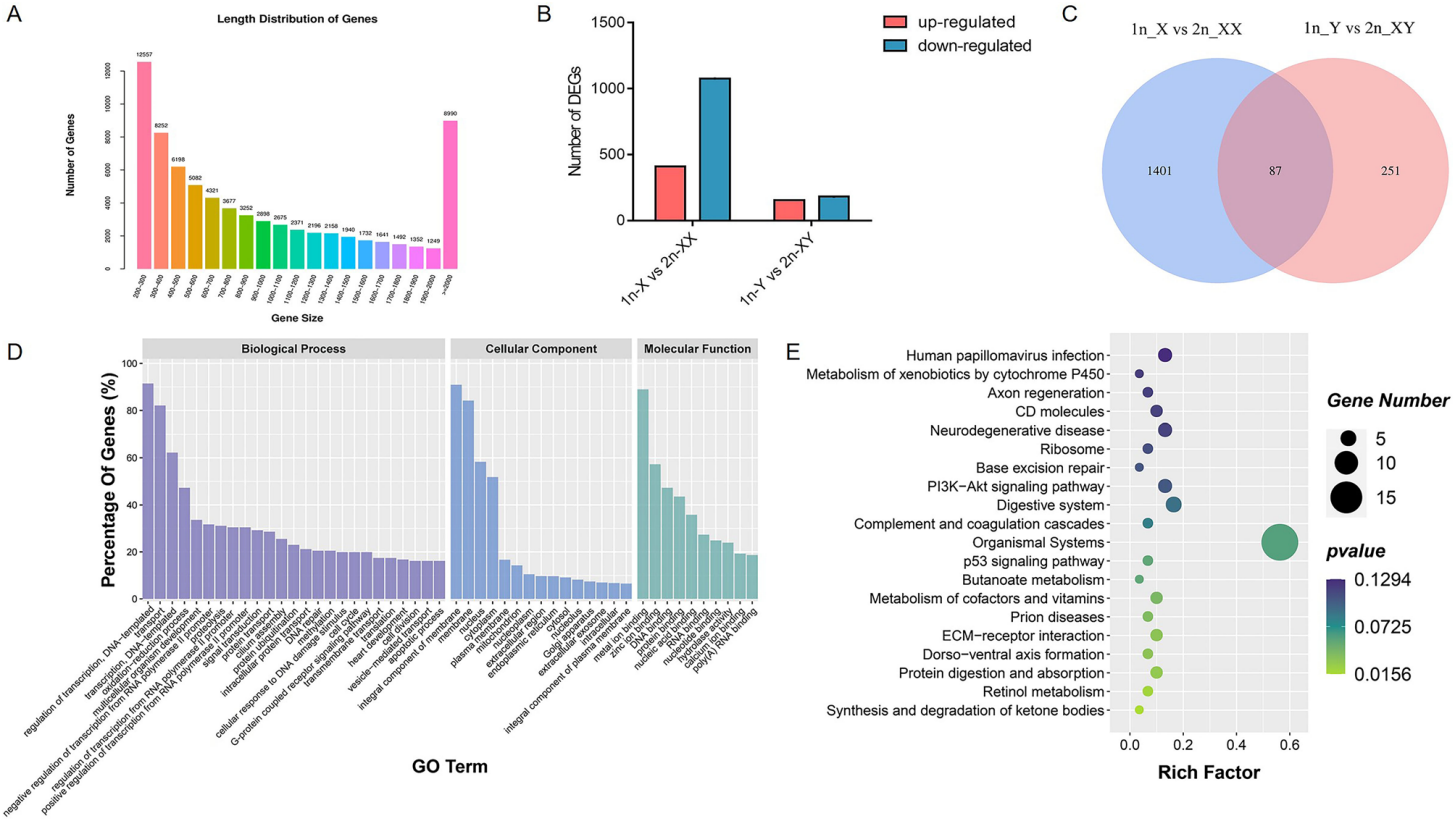
Transcriptome analysis of haploid and diploid tiger pufferfish (*T. rubripes*). (**A**) Length distribution of assembled genes. (**B**) Number of DEGs in 1n-X vs. 2n-XX and 1n-Y vs. 2n-XY comparisons. (**C**) Venn diagram of 1n-X vs. 2n-XX and 1n-Y vs. 2n-XY comparisons. (**D**) GO classification of shared DEGs. (**E**) KEGG enrichment of shared DEGs.

To identify the differentially expressed genes (DEGs) between haploids and diploids and assess the relationship between DNA methylation and gene expression, gene expression profiles were measured by transcriptome sequencing using the same samples in the WGBS study. The data were uploaded to NCBI SRA (SRA accession no.: PRJNA540016). A total of 60.97 G raw bases were obtained. After quality control, a total of 54.00 G clean reads (10.71 G in 1n-X, 13.52 G in 1n-Y, 15.27 G in 2n-XX and 14.50 G in 2n-XY, respectively) with an average Q20 percentage of 97.24% were generated and used for subsequent analysis (Table S5). The de novo assembled transcriptomes included 74,033 unigenes with an N50 length of 1,507 bp and an average length of 716 bp (Fig. 4A, Table S6). The reference transcriptome of tiger pufferfish unigenes was annotated by NCBI, NR, GO, KEGG, Pfam, Swissprot, and EggNOG databases (Table S7). DEG analysis showed that there were 411 DEGs significantly up-regulated and 1077 were down-regulated in 1n-X compared with 2n-XX. Compared with 2n-XY, there were 156 significantly up-regulated and 182 down-regulated DEGs in 1n-Y (Fig. 4B). Venn diagram identified that 87 DEGs were shared both in 1n-X vs. 2n-XX and 1n-Y vs. 2n-XY comparisons (Fig. 4C). To identify transcriptional events occurred during cold-treated haploid induction, DEGs shared in 1n-X vs. 2n-XX and 1n-Y vs. 2n-XY comparisons were subjected to GO enrichment analysis. The GO annotation suggested that regulation of transcription, DNA-templated, transport, and transcription, DNA-templated were the most abundant GO functions in the biological process. Integral component of membrane, membrane, and nucleus were most abundant in the cellular component. In molecular function, GO terms were most enriched for metal ion binding, zinc ion binding, and DNA binding (Fig. 4D). In KEGG enrichment analysis, 153 KEGG pathways were enriched, of which 7 pathways were significantly enriched (*p* < 0.05), including synthesis and degradation of ketone bodies, retinol metabolism, protein digestion and absorption, ECM-receptor interaction, dorsal–ventral axis formation, prion diseases, and metabolism of cofactors and vitamins (Fig. 4E).

To validate the RNA-seq results, relative mRNA levels for six DEGs (*tfap2e*, *olig2*, *prrt1*, *arpp19*, *mafk*, and *wdr1*) were measured by qRT-PCR (Fig. S4). The up- or down-regulation patterns of these genes were consistent in qRT-PCR and RNA-seq results, although few genes showed differentiation between the values. Development related gene *tfap2e* and neuron determination related gene *olig2* were significantly down-regulated in 1n-X vs 2n-XX comparison and have no difference in 1n-Y vs 2n-XY comparison in both qRT-PCR and RNA-seq analysis. Signal transduction related gene *prrt1* was significantly down-regulated in 1n-X vs 2n-XX and 1n-Y vs 2n-XY comparisons in both qRT-PCR and RNA-seq analysis. Mitosis related gene *arpp19* was significantly up-regulated in 1n-X vs 2n-XX comparison and have no difference in 1n-Y vs 2n-XY comparison in both qRT-PCR and RNA-seq analysis. Transcription related gene *mafk* and *wdr1*were significantly down-regulated in 1n-X vs 2n-XX comparison in RNA-seq. While there was down-regulated trend although with no significant difference in 1n-X vs 2n-XX comparison in qRT-PCR analysis.

### Correlation between methylation and gene expression

**Figure 5:**
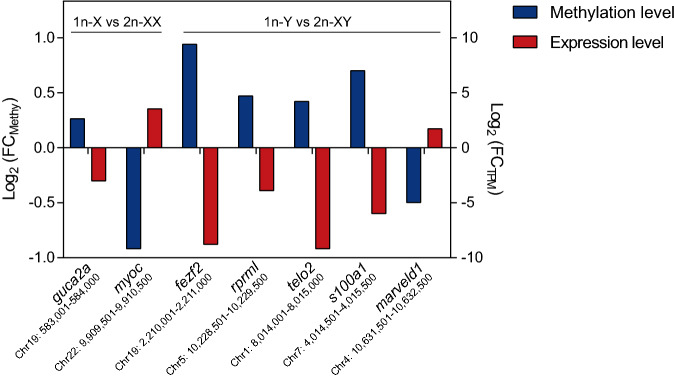
Correlation between methylation and expression levels of target genes. The left Y-axis represents log2 fold change of methylation level of DEGs between haploid and diploid tiger pufferfish, and the right Y-axis represents the log2 fold change of expression level (TPM) of corresponding DEGs between haploid and diploid tiger pufferfish.

In order to analyze the relationship between DNA methylation and DEGs, the whole genome DNA methylation and transcriptional group were combined to screen the genes having a negative relationship between DNA methylation and expression level. The thresholds were set as promoter (TSS − 2.2 kb to + 500 bp) methylation difference > 20%, *q* value < 0.05 for WGBS and log_2_ |fold-change|> 1 and *p* value < 0.05 for transcriptomes. In females, guanylate cyclase activator 2A (*guca2a*) had hyper-methylation level with down-regulated mRNA expression level and myocilin *(myoc*) had hypo-methylation level with up-regulated mRNA expression level in 1n_X vs. 2n_XX comparison. In males, *fez* family zinc finger protein 2 (*fezf2*), reprimo-like protein (*rprml*), telomere length regulation protein TEL2 homolog (*telo2*) and S-100 protein alpha chain (*s100a1*) had hyper-methylation level with down-regulated mRNA expression level, and MARVEL domain-containing protein 1 (*marveld1*) had hypo-methylation level with up-regulated mRNA expression level in 1n_Y vs. 2n_XY comparison (Fig. 5).

## Discussion

Androgenesis is an important manipulation in fish aquaculture for the acquisition of mono-sex fish having sex-biased characteristics and the conservation of fish germplasm^24^. Androgenetic embryos may exhibit haploid syndrome, which includes developmental defects and extensive lethality^25^. DNA methylation is an essential epigenetic mechanism during embryonic development. In order to evaluate methylation and gene expression changes after androgenesis, the whole genome methylation and transcription profiles in haploid and diploid tiger pufferfish were examined. To the best of our knowledge, it is the first WGBS on haploids in aquaculture fish.

We previously established the method of producing androgenetic haploid *T. rubripes* by cold shock treatment, and found that both second polar body and egg nucleus were located on the blastodisc surface in haploid tiger pufferfish embryo, suggesting the simultaneous extrusion of both nuclei^12^. According to our analysis on the haploid and diploid methylome profiles, we found that about 10.80% of C was methylated in both genetic female and male genomes, which is consistent with our previous reports in adult tiger pufferfish^26^ and Chinese tongue sole (*Cynoglossus semilaevis*)^27^. The global DNA methylation level in haploid fish was higher than that in diploid females and males, which may reflect that methylation plays an important role during embryonic development between different ploidy. It also should be noted that although samples used in this study were hatched larva from both haploid and diploid groups, differences in kinetics of development among cell types might still exist. Thus, those developmental differences in cell type distribution might also account for the observed differential DNA methylation. There was limited information on the methylation state in haploid and diploid fish. Therefore, more information on methylation changes among different ploidy levels in fish species is still needed.

Several DMRs were identified between haploid and diploid in both sexes, which indicated that DNA methylation might participate in abnormal development and early embryonic death in haploids. We found GO terms, namely regulation of mitotic cell cycle phase transition and regulation of mitotic sister chromatid segregation, which were enriched. Cell cycle is important for the growth of an organism and plays important roles in cell proliferation, cell-fate decision and many other cell functions^28–30^. According to the previous findings, the duration of mitosis in haploid ESCs is significantly longer than that in diploid embryonic stem cells (ESCs), especially at the metaphase stage^31^. Besides, accelerate G2/M transition could stabilize haploid ESCs in mice^32^. These findings suggest the relationship between the cell cycle and ploidy. In the present study, *slf1* and *actr8* were hyper-methylated in haploid females and males. SLF1 acts with SLF2 as a bridge to link the SMC5/6 complex to RAD18, which is important for higher-order chromatin structure maintenance in mammals. SLF1 depletion can significantly increase abnormalities in anaphase cells^33^. ACTR8 is a key component of the INO80 complex, which has critical functions in DNA replication, repair and recombination, transcription and heterochromatin maintenance^34^. On the other hand, *gas2* and *pbrm1* were hypo-methylated in haploid females and males. GAS2 plays an important role in mitogenesis, cell cycle, microfilament alterations and cellular morphological variation during apoptosis^35–37^. *pbrm1* gene encodes BAF180 protein, which is a subunit of the ATP‐dependent complex SWItch/Sucrose NonFermentable (SWI/SNF) regulating chromatin remodeling^38^. Different methylation levels in these genes between haploid and diploid fish may reflect the effects of ploidy on methylation regulators related to cell cycle and mitosis, which may explain the different development situations between ploidy levels.

Transcriptome analysis revealed subtle alterations in the expression level between haploids and diploids, which was in accordance with the result in *P. olivaceus* as well as mouse embryos^39,40^. KEGG pathways of synthesis and degradation of ketone bodies, retinol metabolism, protein digestion and absorption, ECM-receptor interaction, dorso-ventral axis formation, prion diseases, and metabolism of cofactors and vitamins were significantly enriched (*p* < 0.05) in haploid vs. diploid comparison, indicating that the difference between haploid and diploid is mainly related to biological material and energy metabolism as well as body axis formation. When combined the DNA methylation and transcription data, limited genes showed a negative correlation. *guca2a* gene was hyper-methylated with down-regulated mRNA expression in 1n-X vs. 2n-XX comparison, while *myoc* showed the opposite pattern in this study. It was reported that the down-regulation of GUCA2A was correlated with inflammatory bowel disease and disrupted intestinal homeostasis in mammals^41,42^. Besides, the abnormal intracellular accumulation of myocilin could deteriorate the function of the trabecular meshwork cells and elevate intraocular pressure, which can lead to retinal ganglion cell death^43,44^. In males, *fezf2* and *telo2* were hyper-methylated with down-regulated messenger RNA (mRNA) expression levels in 1n_Y vs. 2n_XY comparison. *fezf2* gene is a highly conserved gene that encodes a zinc finger transcriptional repressor. It is an important regulator to control the development of forebrain neuronal differentiation by promoting local Wnt/β-catenin signaling^45^. TELO2 plays a vital role in the cell cycle by regulating S-phase checkpoint protein. Knockout of *telo2* causes embryonic lethality and S phase cell-cycle arrest in mice^46,47^. *marveld1* gene was hypo-methylated with up-regulated mRNA expression level in 1n_Y vs. 2n_XY comparison. MARVELD1 is a microtubule-associated protein, which plays an important role in cell cycle progression and migration. In mice, overexpression of MARVELD1 can lead to remarkable inhibition of cell proliferation, G1-phase arrest and reduced cell migration^48^. The significant change of these genes indicated abnormal cell cycle, deterioration of visual function and disordered metabolism and development in haploid, which may cause a high fatality rate during early embryonic development. The small fraction of changes in DNA methylation correlate with changes in gene expression also indicated that methylation may affect early embryo development from other ways besides gene expression. It has been reported that DNA methylation can affect genome stability, including chromosome segregation during cell division in mammals^49^. Therefore, the role of DNA methylation in keep chromosome structural stability between different ploidy worth exploring in future research.

## Conclusion

Our study revealed different methylation and expression profiles between haploid and diploid teleost for the first time. Several genes were identified showing different performances between different ploidy levels that may participate in haploid syndrome formation. These results may expand the understanding of the effects of ploidy on early development in teleost and provide knowledge about target genes and networks to improve the survival rate of haploids. Moreover, future studies are needed to determine the roles of these target genes during early embryonic development.

## Supplementary Information

Supplementary Figure S1.

Supplementary Figure S2.

Supplementary Figure S3.

Supplementary Figure S4.

Supplementary Table S1.

Supplementary Table S2.

Supplementary Table S3.

Supplementary Table S4.

Supplementary Table S5.

Supplementary Table S6.

Supplementary Table S7.

Supplementary Information 12.
