# Supplementary figures and images for: Genome-wide DNA methylation and gene expression patterns of androgenetic haploid tiger pufferfish (Takifugu rubripes) provide insights into haploid syndrome
Source: Sci Rep. 2022 May 18;12:8252. doi: 10.1038/s41598-022-10291-z (PMC9117679; doi:10.1038/s41598-022-10291-z)

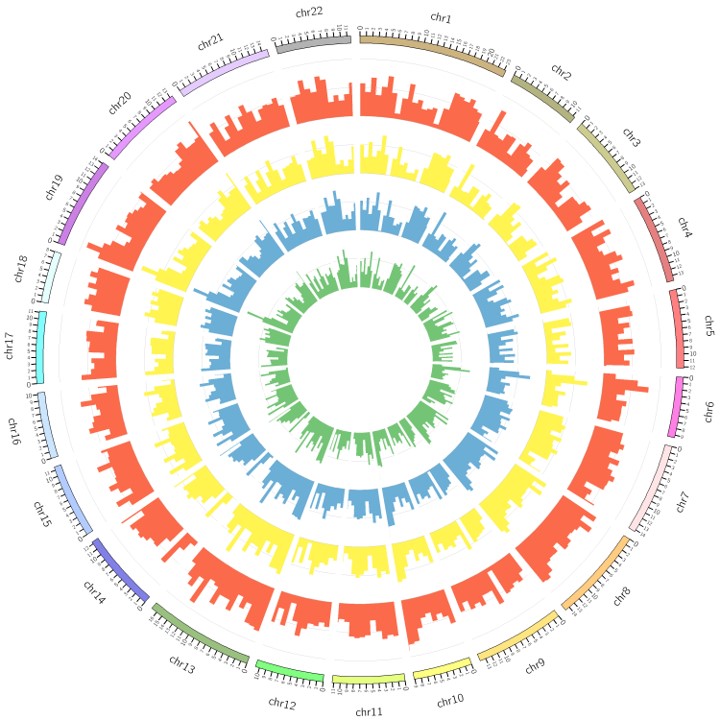

Supplement: Supplementary file 1 — Supplementary Figure S1. [file 41598_2022_10291_MOESM1_ESM.jpg]

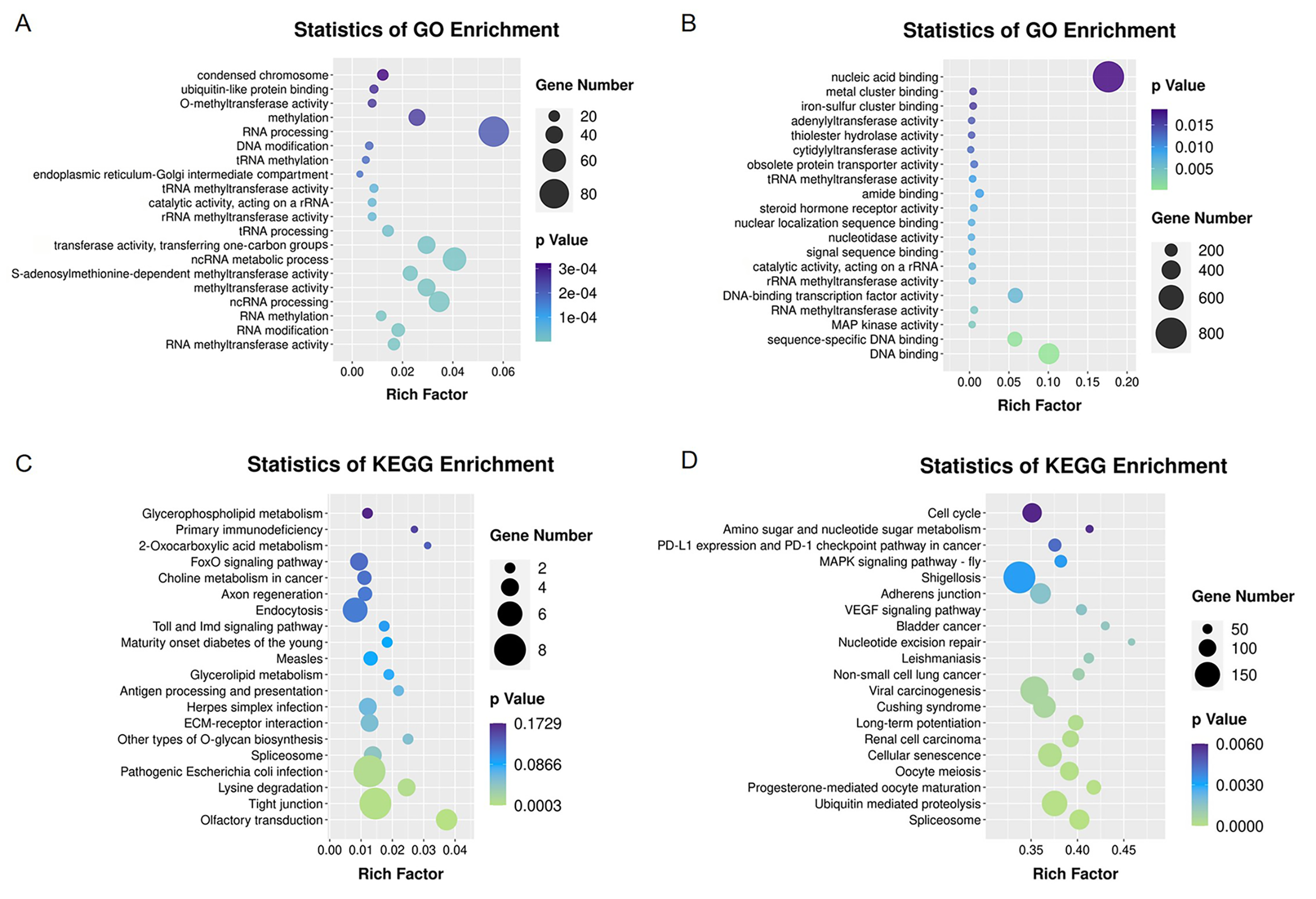

Supplement: Supplementary file 2 — Supplementary Figure S2. [file 41598_2022_10291_MOESM2_ESM.tif]

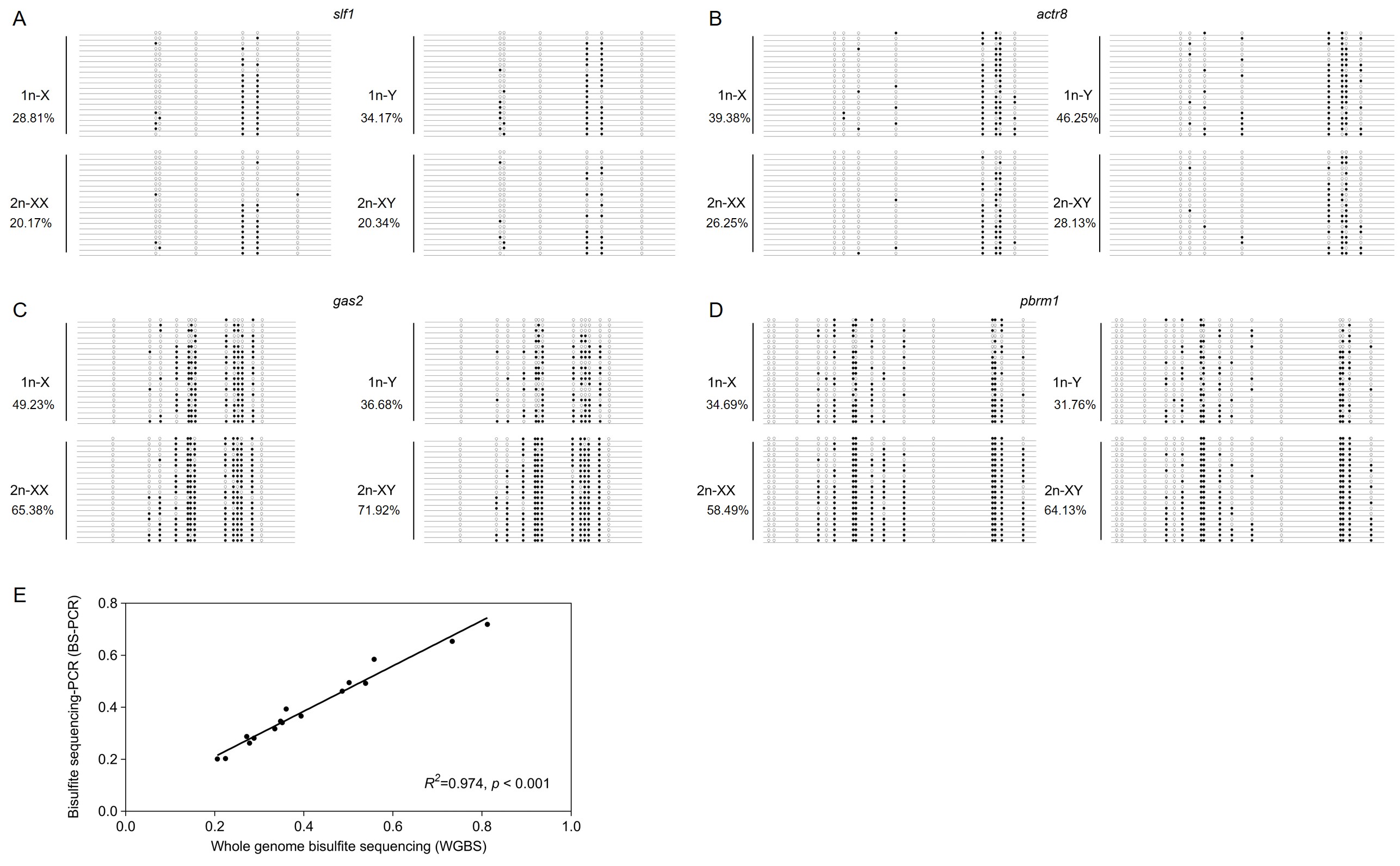

Supplement: Supplementary file 3 — Supplementary Figure S3. [file 41598_2022_10291_MOESM3_ESM.jpg]

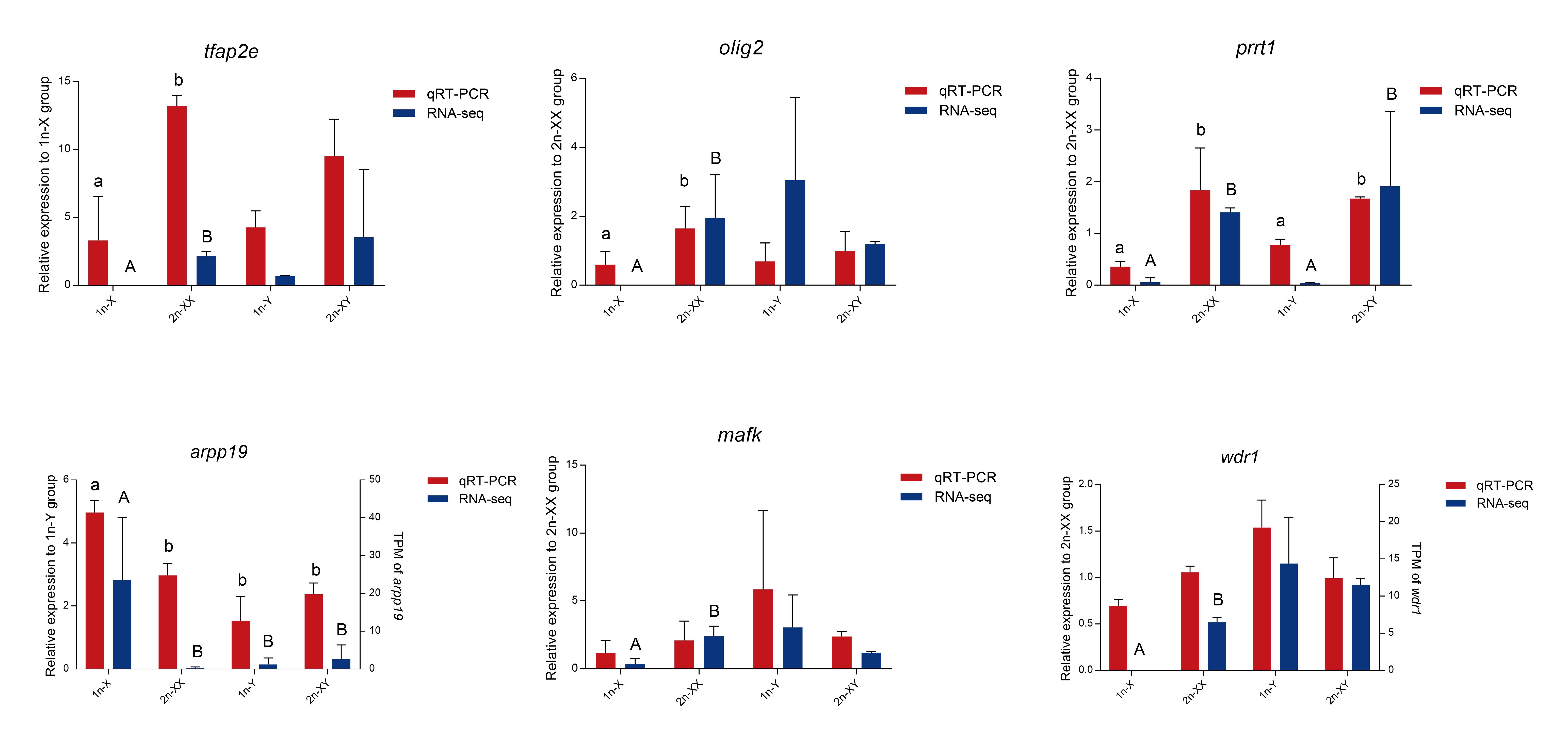

Supplement: Supplementary file 4 — Supplementary Figure S4. [file 41598_2022_10291_MOESM4_ESM.jpg]
