# Supplementary material for: Genome-wide DNA methylation and gene expression patterns of androgenetic haploid tiger pufferfish (Takifugu rubripes) provide insights into haploid syndrome
Source: Sci Rep. 2022 May 18;12:8252. doi: 10.1038/s41598-022-10291-z (PMC9117679; doi:10.1038/s41598-022-10291-z)
Supplement: Supplementary file 5 — Supplementary Table S1. [file 41598_2022_10291_MOESM5_ESM.docx]

**Table S1.** The primers used in this study.

| Primer name | Sequence (5’-3’) | Usage |
| --- | --- | --- |
| *slf1*-F | ATATTGTTTATATTGTTATTATTGTAAATT | BS-PCR |
| *slf1*-R | TAAAACAAAACTCTCTTCTTTTCAC |  |
| *actr8*-F | GGTTTTGGAGGATTGAATATAAAATAG | BS-PCR |
| *actr8*-R | AATTAACCCAAACTTAACAAAAAAC |  |
| *gas2*-F | TATTATTGGTTGTTTTGTTTTTTTT | BS-PCR |
| *gas2*-R | ACCCCAATCTACTATCCTACAAAAC |  |
| *pbrm1*-F | TGTTGATATTTTGTTTTTATTTTAA | BS-PCR |
| *pbrm1*-R | AACTACAAAACTTCTTACAAAACC |  |
| *tfap2e*-qF | CCTTATCCGCAATCGTCTCTG | qRT-PCR validation |
| *tfap2e*-qR | ATGTTGATGGATGGAGTTGATGG |  |
| *olig2*-qF | ACTCGCTGGAAGAGATGAAG | qRT-PCR validation |
| *olig2*-qR | GGTGTGAGCTGTGAGATGTG |  |
| *prrt1*-qF | ACGACCGTCTGTTGCTTCTG | qRT-PCR validation |
| *prrt1*-qR | GCCAGGCTGATGAAGGAGAA |  |
| *arpp19*-qF | CCGAGGAGATTGAAGGAACC | qRT-PCR validation |
| *arpp19*-qR | CCGCTTTCTCTGGACTGATG |  |
| *mafk*-qF | GTGTCACGCAGAAGGAGGAG | qRT-PCR validation |
| *mafk*-qR | CTCATGCTGGCGTTCTCTCG |  |
| *wdr1*-qF | AGACCAAGCATTACCAGAAAGTG | qRT-PCR validation |
| *wdr1*-qR | GAGACTACGGCATCCTGAAGA |  |
| *amhr2*-F | ACGATGCACACAAACCACCT | Genetic sex identification |
| *amhr2*-R | TCCCAGTGTTGCGGTATGTA |  |
