# Supplementary material for: Genome-wide DNA methylation and gene expression patterns of androgenetic haploid tiger pufferfish (Takifugu rubripes) provide insights into haploid syndrome
Source: Sci Rep. 2022 May 18;12:8252. doi: 10.1038/s41598-022-10291-z (PMC9117679; doi:10.1038/s41598-022-10291-z)
Supplement: Supplementary file 6 — Supplementary Table S2. [file 41598_2022_10291_MOESM6_ESM.docx]

**Table S2.** Statistic of WGBS-seq for each sample.

| Sample | Clean reads | Clean bases  (G) | Q20% | Unique mapped reads | Unique  mapped  rate (%) | Usable depth (X/strand) | C coverage (%) |
| --- | --- | --- | --- | --- | --- | --- | --- |
| 1n-X_1 | 73891658 | 11.08 | 89.71 | 33078348 | 47.67 | 29.97 | 34.19 |
| 1n-X_2 | 73628012 | 11.05 | 85.91 | 33792739 | 45.90 | 29.86 | 32.48 |
| 1n-Y_1 | 78550728 | 11.78 | 87.72 | 26242053 | 36.74 | 31.86 | 31.11 |
| 1n-Y_2 | 88574380 | 13.29 | 89.72 | 38972144 | 46.34 | 35.92 | 35.24 |
| 2n-XX_1 | 75874088 | 11.38 | 89.62 | 35168151 | 47.90 | 30.77 | 35.25 |
| 2n-XX_2 | 101072246 | 15.16 | 85.12 | 22227499 | 25.29 | 40.99 | 27.31 |
| 2n-XY_1 | 76103864 | 11.42 | 90.45 | 38051851 | 51.42 | 30.86 | 35.35 |
| 2n-XY_2 | 74420212 | 11.16 | 91.58 | 41739489 | 57.42 | 30.18 | 35.37 |
