# Supplementary material for: Genome-wide DNA methylation and gene expression patterns of androgenetic haploid tiger pufferfish (Takifugu rubripes) provide insights into haploid syndrome
Source: Sci Rep. 2022 May 18;12:8252. doi: 10.1038/s41598-022-10291-z (PMC9117679; doi:10.1038/s41598-022-10291-z)
Supplement: Supplementary file 7 — Supplementary Table S3. [file 41598_2022_10291_MOESM7_ESM.docx]

**Table S3.** Percentages of mC in the mCG, mCHG, and mCHH contexts.

| Sample | mC | mCG | mCG (%) | mCHG | mCHG （%） | mCHH | mCHH (%) |
| --- | --- | --- | --- | --- | --- | --- | --- |
| 1n-X_1 | 104546177 | 95493348 | 91.34% | 2574716 | 2.46% | 6478113 | 6.20% |
| 1n-X_2 | 101636170 | 93724067 | 92.22% | 2330806 | 2.29% | 5581297 | 5.49% |
| 1n-Y_1 | 84763379 | 73917819 | 87.20% | 2997840 | 3.54% | 7847720 | 9.26% |
| 1n-Y_2 | 122561037 | 109199176 | 89.10% | 3759646 | 3.07% | 9602215 | 7.83% |
| 2n-XX_1 | 105851520 | 97873297 | 92.46% | 2301700 | 2.17% | 5676523 | 5.36% |
| 2n-XX_2 | 96837959 | 89599099 | 92.52% | 2106894 | 2.18% | 5131966 | 5.30% |
| 2n-XY_1 | 113281727 | 103959001 | 91.77% | 2677357 | 2.36% | 6645369 | 5.87% |
| 2n-XY_2 | 123914637 | 112715537 | 90.96% | 3219796 | 2.60% | 7979304 | 6.44% |
