# Supplementary material for: Genome-wide DNA methylation and gene expression patterns of androgenetic haploid tiger pufferfish (Takifugu rubripes) provide insights into haploid syndrome
Source: Sci Rep. 2022 May 18;12:8252. doi: 10.1038/s41598-022-10291-z (PMC9117679; doi:10.1038/s41598-022-10291-z)
Supplement: Supplementary file 9 — Supplementary Table S5. [file 41598_2022_10291_MOESM9_ESM.docx]

**Table S5.** Summary of RNA sequencing of tiger pufferfish (*T. rubripes*).

| Sample | Raw Reads | Raw Bases | Valid Bases | Valid% | Q20% | Q30% | GC% |
| --- | --- | --- | --- | --- | --- | --- | --- |
| 1n-X-1 | 32543750 | 4.91 G | 3.36 G | 72.03 | 96.30 | 92.24 | 46.31 |
| 1n-X-2 | 54985602 | 8.30 G | 7.35 G | 92.72 | 97.03 | 93.55 | 47.83 |
| 1n-Y-1 | 49100048 | 7.41 G | 6.79 G | 95.02 | 97.55 | 94.22 | 49.39 |
| 1n-Y-2 | 51143950 | 7.72 G | 6.73 G | 90.82 | 97.29 | 93.84 | 48.13 |
| 2n-XX-1 | 52496556 | 7.93 G | 7.29 G | 95.24 | 97.68 | 94.49 | 49.78 |
| 2n-XX-2 | 57456148 | 8.68 G | 7.98 G | 96.04 | 97.52 | 94.26 | 49.85 |
| 2n-XY-1 | 57530998 | 8.69 G | 7.90 G | 94.85 | 97.30 | 93.90 | 49.04 |
| 2n-XY-2 | 48550586 | 7.33 G | 6.60 G | 93.91 | 97.23 | 93.76 | 48.81 |
