# Supplementary material for: Genome-wide DNA methylation and gene expression patterns of androgenetic haploid tiger pufferfish (Takifugu rubripes) provide insights into haploid syndrome
Source: Sci Rep. 2022 May 18;12:8252. doi: 10.1038/s41598-022-10291-z (PMC9117679; doi:10.1038/s41598-022-10291-z)
Supplement: Supplementary file 10 — Supplementary Table S6. [file 41598_2022_10291_MOESM10_ESM.docx]

**Table S6.** Assembly statistics of RNA sequencing of tiger pufferfish (*T. rubripes*).

| Index | Number | GC% | Min Length/bp | Median Length/bp | Max Length/bp | Total Assembled Bases | N50/bp |
| --- | --- | --- | --- | --- | --- | --- | --- |
| Transcript | 91717 | 49.19 | 201 | 731 | 10795 | 92129003 | 1507 |
| Gene | 74033 | 49.08 | 201 | 716 | 10795 | 73592411 | 1507 |
