# Supplementary material for: Genome-wide DNA methylation and gene expression patterns of androgenetic haploid tiger pufferfish (Takifugu rubripes) provide insights into haploid syndrome
Source: Sci Rep. 2022 May 18;12:8252. doi: 10.1038/s41598-022-10291-z (PMC9117679; doi:10.1038/s41598-022-10291-z)
Supplement: Supplementary file 11 — Supplementary Table S7. [file 41598_2022_10291_MOESM11_ESM.docx]

**Table S7.** Annotation statistics of RNA sequencing of tiger pufferfish (*T. rubripes*).

| Database | No. | Ratio (%) |
| --- | --- | --- |
| All | 74033 | 100.00 |
| GO | 22604 | 30.53 |
| KEGG | 14350 | 19.38 |
| Pfam | 19274 | 26.03 |
| Swissprot | 21000 | 28.37 |
| EggNOG | 25327 | 34.21 |
| NR | 27019 | 36.50 |
