# Supplementary material for: Genome-wide DNA methylation and gene expression patterns of androgenetic haploid tiger pufferfish (Takifugu rubripes) provide insights into haploid syndrome
Source: Sci Rep. 2022 May 18;12:8252. doi: 10.1038/s41598-022-10291-z (PMC9117679; doi:10.1038/s41598-022-10291-z)
Supplement: Supplementary file 12 — Supplementary Information 12. [file 41598_2022_10291_MOESM12_ESM.docx]

**Supplementary Material**

**Figure S1.** Distribution of mCG at the chromosome level in haploid and diploid tiger pufferfish. The red, yellow, blue, and green lines represent haploid female (1n-X) and male (1n-Y), and diploid female (2n-XX), and male (2n-XY), respectively.

**Figure S2.** GO and KEGG pathway analyses of DMGs between haploid and diploid tiger pufferfish (*T. rubripes*). (A) Enriched GO terms between haploid and diploid females. (B) Enriched GO terms between haploid and diploid males. (C) Enriched KEGG pathways between haploid and diploid females. (D) Enriched KEGG pathways between haploid and diploid males. Top 20 terms and pathways are shown. In C and D, duplicate pathway names from different species are represented by one.

**Figure S3.** Validation of DNA methylation data by BS-PCR in haploid and diploid tiger pufferfish. (A-D) Four DEGs, including *slf1*, *actr8*, *gas2*, and *pbrm1* were used for BS-PCR. The filled or open circles indicate methylated or unmethylated CpG sites, respectively, and each row represents one sequenced clone. (E) Dot plot showing correlation between whole genome bisulfite sequencing (WGBS) and bisulfite sequencing-PCR (BS-PCR).

**Figure S4.** Validation of RNA-Seq data by qRT-PCR. Six DEGs, including *tfap2e*, *olig2*, *prrt1*, *arpp19*, *mafk*, and *wdr1* were used for qRT-PCR. The left Y-axis represents the relative expression of DEGs. For *arpp19* and *wdr1*, the right Y-axis represents TPM by RNA-Seq. Different letters indicate statistically significant differences (*p* < 0.05).

**Table S1.** The primers used in this study.

**Table S2.** Statistic of WGBS-seq for each sample.

**Table S3.** Percentages of mC in the mCG, mCHG, and mCHH contexts.

**Table S4.** Enriched GO biological process of haploid hyper-methylated and haploid hypo-methylated DMGs shared in female and male tiger pufferfish (*T. rubripes*).

**Table S5.** Summary of RNA sequencing of tiger pufferfish (*T. rubripes*).

**Table S6.** Assembly statistics of RNA sequencing of tiger pufferfish (*T. rubripes*).

**Table S7.** Annotation statistics of RNA sequencing of tiger pufferfish (*T. rubripes*).
